# Mediterranean diet: the impact on cardiovascular risk and metabolic syndrome in HIV patients, in Lisbon, Portugal

**DOI:** 10.7448/IAS.17.4.19727

**Published:** 2014-11-02

**Authors:** Sara Policarpo, Emilia Valadas, Teresa Rodrigues, Ana Catarina Moreira

**Affiliations:** 1 Nutrition and Dietetics Department, University Hospital of Santa Maria, Lisbon, Portugal; 2 Infectious Diseases Department, University Hospital of Santa Maria, Lisbon, Portugal; 3 Laboratory of Biomathematics, Faculty of Medicine of the University of Lisbon, Lisbon, Portugal; 4 Dietetics Department, Lisbon School of Health Technology, Lisbon, Portugal

## Abstract

**Introduction:**

Metabolic syndrome (MS) is common in HIV-infected individuals and it is associated with higher cardiovascular risk (CVR). Mediterranean diet has been associated with a better metabolic control and lower CVR.

**Materials and Methods:**

From December 2013 to May 2014, individuals between 18 and 65 years of age, who attended the outpatient HIV Clinic at the University Hospital Santa Maria, Lisbon, were selected. Adherence to Mediterranean diet was evaluated with MedDietScore, a scale from 0 to 55 that punctuates 11 food items according to the frequency of intake. Higher scores represent higher adherence. CVR was assessed using D.A.D tool (classified as low, moderate or high risk). We excluded individuals with opportunistic disease, hospitalized in the past three months or with renal disease diagnosis. All participants gave written informed consent.

**Results:**

In the 571 HIV patients included, 67.1% (n=383) were male, 91.6% (n=523) Caucasian, with a mean age of 46.5±8.9 years. Patients were divided in two groups: naïve (7.5%; n=43) or on antiretroviral treatment (ART) (92.5%; n=528). Mean length of HIV diagnosis was 6.7±6.5 years (naïve) and 13.3±6.1 years (ART); TCD4+ counts were above 500 cel/mm3 in 55.8% (n=24) and 67.6% (n=357) of the patients, respectively. MS was present in 33.9% (n=179) of patients in ART group and 16.3% (n=7) in naïve group. Presence of MS was associated with ART group (OR=2.7; p=0.018). MS was also associated with older age in this group (p=0.000). Overall, mean MedDietScore was 27.3±5.5. Higher score was associated with older age (r=0.319; p=0.000). Naïve patients presented a trend to higher adherence to Mediterranean diet (65.1% vs 51.7% in naïve group; p=0.090). No relation between MS and Mediterranean diet was found. Higher CVR was associated with the presence of MS in the ART group (p=0.001). In this group, individuals with moderate CVR presented higher rates of adherence to Mediterranean diet (p=0.036) when compared to low and high CVR score.

**Conclusions:**

In this cross-sectional study, naïve individuals presented a trend to higher adherence to Mediterranean diet. On the ART group, higher adherence to Mediterranean diet was found in individuals with moderate CVR score. We think that this might suggest that this group of patients adopt this diet only in the presence of metabolic alterations or perceived CVR. Prospective studies in HIV patients are required to determine the impact of adherence to Mediterranean diet on the reduction of CVR.

